# Cytokine and Antibody Responses to *Plasmodium falciparum* in Naïve Individuals during a First Malaria Episode: Effect of Age and Malaria Exposure

**DOI:** 10.1371/journal.pone.0055756

**Published:** 2013-02-21

**Authors:** Gemma Moncunill, Alfredo Mayor, Alfons Jiménez, Augusto Nhabomba, Laura Puyol, Maria N. Manaca, Diana Barrios, Pau Cisteró, Caterina Guinovart, Ruth Aguilar, Azucena Bardají, María-Jesús Pinazo, Evelina Angov, Sheetij Dutta, Chetan E. Chitnis, José Muñoz, Joaquim Gascón, Carlota Dobaño

**Affiliations:** 1 Barcelona Centre for International Health Research (CRESIB, Hospital Clínic-Universitat de Barcelona), Barcelona, Spain; 2 Centro de Investigação em Saúde de Manhiça, Maputo, Mozambique; 3 Walter Reed Army Institute of Research (WRAIR), Silver Spring, Maryland, United States of America; 4 International Centre for Genetic Engineering and Biotechnology (ICGEB), New Delhi, India; Burnet Institute, Australia

## Abstract

Age- and exposure-dependent immune responses during a malaria episode may be key to understanding the role of these factors in the acquisition of immunity to malaria. Plasma/serum samples collected from naïve Mozambican children (n = 48), European adults (naïve travelers, n = 22; expatriates with few prior malaria exposures, n = 15) and Mozambican adults with long-life malaria exposure (n = 99) during and after a malaria episode were analyzed for IgG against merozoite proteins by Luminex and against infected erythrocytes by flow cytometry. Cytokines and chemokines were analyzed in plasmas/sera by suspension array technology. No differences were detected between children and adults with a primary infection, with the exception of higher IgG levels against 3D7 MSP-1_42_ (*P* = 0.030) and a *P. falciparum* isolate (*P* = 0.002), as well as higher IL-12 (*P* = 0.020) in children compared to other groups. Compared to malaria-exposed adults, children, travelers and expatriates had higher concentrations of IFN-γ (*P≤*0.0090), IL-2 (*P≤*0.0379) and IL-8 (*P≤*0.0233). Children also had higher IL-12 (*P* = 0.0001), IL-4 (*P* = 0.003), IL-1β (*P* = 0.024) and TNF (*P* = 0.006) levels compared to malaria-exposed adults. Although IL-12 was elevated in children, overall the data do not support a role of age in immune responses to a first malaria episode. A T_H_1/pro-inflammatory response was the hallmark of non-immune subjects.

## Introduction

The worldwide burden of disease from malaria remains a major public health problem, with approximately 216 million cases and 655,000 deaths in 2010, with the majority of deaths occurring in sub-Saharan Africa [1]. In areas where malaria is endemic, children together with pregnant women are the most susceptible populations. Children suffer more malaria episodes and are more prone to severe malaria compared to adults [2]. With increasing age, there is a shift from severe malaria to clinical malaria and eventually to asymptomatic parasitemia, but immunity is never sterilizing. Malaria exposure but also age may have an effect on the natural acquisition of immunity as the immune system of children is not totally mature [3], [4]. After several decades of investigation in animal models and humans, the mechanisms involved in naturally acquired immunity and immunopathology are still poorly defined, partly because it is difficult to separate the effects of age from those of repetitive exposure. There is no clear evidence for an effect of age at first exposure to *Plasmodium falciparum* in the development of immunity in African children [5], [6], although previous studies in malaria – naïve migrants in Indonesia suggested that the more mature immune system from an adult may allow acquisition of immunity more rapidly than the less mature immune system from a child under the same conditions of exposure [7], [8]. Totally naïve adults such as travelers or migrants never exposed to *P. falciparum* going to malaria-endemic areas are also more susceptible to clinical and severe malaria than adults from endemic areas [2], [9], [10]. Furthermore, it has been suggested that naïve adults are initially more susceptible to severe disease than children [2], [11] and show different clinical presentations [12].

In this study, we aimed to determine (i) the effect of age on cytokine, chemokine and Immunoglobulin G (IgG) responses in children and adults during the acute and convalescent phases of a first *P. falciparum* malaria episode and (ii) the effect of exposure on immune responses in adults during the acute and convalescent phases of a malaria episode. We hypothesized that the immune response of children during a first malaria episode would be quantitatively and qualitatively different from the immune response of naïve adults. Further, we hypothesized that during a malaria episode the immune response in naïve adults would be quantitatively and qualitatively different from immune responses in adults with different levels of prior exposure to *P. falciparum* infection.

## Materials and Methods

### Ethics Statement

Written informed consent was obtained from participants or their respective parents or guardians before sample collection. Parasitemic individuals were treated according to standard national guidelines at the time of the studies. Approval for the protocols was obtained from the National Mozambican Ethics Review Committee and the Hospital Clínic of Barcelona Ethics Review Committee.

### Study design, patients and sample collection

All volunteers included in this study had an acute clinical malaria episode at the moment of recruitment and/or sample collection. There were four groups of patients: (i) naïve children from a malaria endemic area with a first episode (ii) naïve adults from a non-endemic area with a first episode (travelers) (iii) other non-immune adults temporarily resident in an endemic area (expatriates) and (iv) adults from an endemic area (malaria-exposed).

Children were recruited in the context of a study conducted at the Centro de Investigação em Saúde de Manhiça, Manhiça District, southern Mozambique, where transmission of *P. falciparum* is perennial, with some seasonality of moderate intensity. Forty eight children presenting a first malaria episode were chosen from a total of 287 followed up from birth to age 24 months in a three-arm randomized, double-blind, placebo-controlled trial with monthly chemoprophylaxis with sulfadoxine-pyrimethamine and artesunate between 2005 and 2009 [5]. Children had been followed up by a combination of active and passive case detection. Blood samples were collected into EDTA microtainers by finger-prick at acute episode (day 0) and at convalescence after treatment (day 28). Two blood smears and blood spots onto filter paper (Schleicher and Schuell; no. 903TM) were used to determine parasitemia by microscopy [5] and PCR, respectively.

Travelers and expatriates were European adults without previous malaria exposure (22 travelers) or who have lived in a malaria endemic area for a minimum of one year (15 expatriates). They were recruited at the Tropical Medicine Unit in the Hospital Clínic de Barcelona, Spain, between 2005 and 2009, and diagnosed with *P. falciparum* malaria by microscopy after traveling to an African country. Blood samples from acute episodes (day 0) and convalescence after malaria treatment (days 7 and 28) were collected by venipuncture into one heparin vacutainer for infected erythrocyte (IE) pellet cryopreservation in glycerolyte solution, and one vacutainer without anticoagulant for serum cryopreservation at −80°C. Two blood drops from each sample were spotted onto filter paper for parasite PCR analysis.

Malaria-exposed adults with life-long exposure to *P. falciparum* were non-pregnant women and men recruited from patients attending the Manhiça District Hospital, Mozambique, with clinical malaria between 2004 and 2005 [13]. Blood samples were collected by venipuncture into heparin tubes and plasma samples were cryopreserved at −80°C. Two blood drops from each sample were spotted onto filter paper for parasite PCR detection.

### Recombinant proteins

Apical membrane antigen 1 (AMA-1) from the 3D7 strain [14], the receptor-binding region F2 of the 175 kDa erythrocyte binding antigen from the CAMP strain (EBA-175; F2 region) [15], the Duffy binding-like alpha (DBL-α) domain of *Pf*EMP-1 [16] and the DBL3X domain of VAR2CSA *Pf*EMP-1 [17] were produced as recombinant proteins at ICGEB. AMA-1 from FVO strain and MSP-1_42_ from 3D7 and FVO strains [18], [19] were produced as recombinant proteins at WRAIR.

### Antibody levels to recombinant proteins

A multiplex suspension array technology panel was established to quantify IgG responses to *P. falciparum* antigens using Luminex xMAP^TM^ (Luminex Corp., Austin, Texas) and the Bio-Plex 100 platform (Bio-Rad, Hercules, California) [20]. Briefly, 3,000 microspheres per analyte were used per sample test. A pool from hyper-immune Mozambican adult volunteers at dilution 1/30,000 and samples from non-exposed European adults at dilution 1/1,000 were added in duplicates to each plate as positive and negative controls, respectively. Study samples were tested in duplicates at dilutions 1/1,000 and 1/30,000, but only 1/1,000 was chosen for the statistical analysis because of a wider quantitative dynamic range. In some wells no serum/plasma was added as a control of background. Beads were incubated with serum/plasma samples for 1 h. The plate was washed by pelleting microspheres (centrifuge at 800 *g* for 5 min) and resuspended with 0.05% Tween 20-PBS. One hundred µL of biotinylated anti-human IgG (Sigma, Tres Cantos, Spain) diluted 1/2,500 in assay buffer were applied and incubated for 45 min. The plate was washed as before, and 100 µL of streptavidin-conjugated R-phycoerythrin (Invitrogen, Carlsbad, California) diluted 1/1,000 in assay buffer were added and incubated for 25 min. All incubations were done at room temperature (RT) with plate agitation and protection from light. The plate was read using Bio-Plex Manager version 4.0, and at least 100 microspheres per analyte were acquired per sample. Mean fluorescent intensity (MFI) with background fluorescence subtracted was exported and arbitrary units (AU) concentration for each antigen was calculated by dividing MFI of each sample by the MFI of the positive control run in each plate.

### Antibodies to IEs surface antigens

Two laboratory clones (CS2, R29), 3 isolates collected from travelers to endemic regions (IE_Trav1_, IE_Trav2_, IE_Trav3_), 2 pediatric isolates from Mozambican children, one with uncomplicated malaria (IE_Ch1_) and another with severe malaria (IE_Ch2_) and 1 isolate from a pregnant Mozambican woman (IE_Woman_), each of whom had the O blood group, were tested for recognition by IgG using flow cytometry. Study samples were tested blindly in a single assay against each parasite. A pool of plasma samples from immune Mozambican adults and a pool of plasma samples from non-exposed European adults were included as positive and negative controls, respectively. Cryopreserved ring-stage parasites were thawed in a sorbitol gradient and grown to late trophozoites. Cells were washed 3 times in PBS and resuspended at 1% hematocrit and 1–5% parasitemia in 1% BSA-PBS solution. Five μL of plasma were added to 95 μL/well of erythrocyte suspension, incubated for 30 min at RT and stained for 30 min with 100 μL of polyclonal rabbit anti-human IgG (DakoCytomation) at 1/200. Subsequently, cells were incubated with 100 μL of AlexaFluor®-conjugated donkey anti-rabbit IgG (Invitrogen) diluted at 1/1,000 and 10 µg/mL of ethidium bromide (Ecogen) for 30 min in darkness at RT. Samples were washed 3 times with PBS-BSA between incubations. Data from 1,000 positive events was acquired with a Becton-Dickinson (BD) FACSCalibur flow cytometer. Reactivity against IE surface antigens was expressed as the difference between the MFI of IEs and the MFI of uninfected erythrocytes.

### Cytokine/chemokine concentrations in plasma/serum

Concentrations (pg/mL) of interleukin (IL)-12p70, IL-2, IFN-γ, IL-4, IL-5, IL-10, IL-8, IL-6, IL-1β, TNF and TNF-β in plasma and serum were measured using a commercial multiplex suspension array technology kit (Human Th1/Th2 11plex FlowCytomix kit, Bender MedSystems) and flow cytometry. Twenty-five µL of plasma or serum were tested following manufacturer’s instructions. MFI from microspheres was acquired with a BD FACSCanto II and analyzed in FlowCytomix Pro2.2.1 software (Bender MedSystems). Concentration of each analyte was obtained by interpolating fluorescence intensity to a 7-point dilution standard curve supplied by the manufacturer. Any value below the limits of detection was given a value of half the detection limit for that cytokine. TNF-β was excluded from the statistical analysis since levels in most samples (226/233, 97%) were below the limit of detection.

### Statistical methods

Recognition of recombinant proteins and parasites by antibodies was considered positive if AU or MFI values were above the mean of the negative controls plus 2 standard deviations for each antigen. Threshold values for seroprevalence were: 238.02 AU for AMA-1 3D7; 1134.73 AU for AMA-1 FVO; 921.18 AU for MSP-1_42_ 3D7; 638.33 AU for MSP-1_42_ FVO; 3110.36 AU for EBA-175; 1572.55 AU for DBL-α; and 217.44 AU for DBL3X. Comparisons between groups for categorical variables were done using χ^2^test or Fisher’s exact test. Continuous variables were analyzed using the non-parametric Kruskal Wallis test or the Mann-Whitney U test. Correlations within groups were assessed by Spearman’s rank coefficient. All *p*-values were two-sided and considered statistically significant when <0.05. All data collected were analyzed using Stata version 11.0 (Stata Corporation, College Station, Texas, USA).

## Results

### Description of participants

Table 1 details the characteristics of the study subjects. There were significant differences in the median age between children, travelers, expatriates and malaria-exposed individuals and no significant differences in the gender distribution. Children with no documented prior malaria episodes and malaria-exposed adults were from malaria endemic areas of Mozambique, whereas travelers and expatriates were from non-malaria endemic countries. Travelers had never experienced malaria before, whereas expatriates had lived in malaria endemic areas and 12 out of 15 reported at least one previous malaria episode. Malaria-exposed adults were individuals living in *P. falciparum* malaria endemic regions presenting with an episode of clinical malaria. There were no differences in parasite densities between travelers and expatriates and between children and malaria-exposed adults. Some children were born to mothers with placental malaria infection defined as having malaria pigment or parasites in the placenta (10 out of 38 mothers, 10 unknown) and 3 children had cord blood that was PCR positive for *P. falciparum*.

**Table 1:** Characteristics of the study participants.

Characteristics	Children	Travelers	Expatriates	Malaria-exposed	*P-value*
Day 0, n	48	22	15	99	
Day 7, n	0	12	10	0	
Day 28, n	28	6	4	0	
Age, median years (IQR)	1.1 (0.9; 1.4)	31 (28; 38)	53 (45; 60)	26 (19; 36)	*<0.001* a
Male, n (%)	26 (54)	15 (75)	7 (58)	52 (53)	*0.320* b
Origin, n (%)					
African	48 (100)	0 (0)	0 (0)	99 (100)	
European	0 (0)	17 (85)	15 (100)	0 (0)	
Others	0 (0)	3 (15)	0 (0)	0 (0)	
Previous malaria episodes, n (%)	0 (0)	0 (0)	12 (80)	99 (100) c	*<0.001* b
Parasitemia by thin smear microscopy, %	ND	0.08 (0.01; 0.80)	0.06 (0.01; 0.19)	ND	*0.888* d
Parasitemia by thick smear microscopy, parasites/μl e	26032 (9608; 45926)	ND	ND	35379 (14338; 61176)	*0.091* d

Abbreviations: IQR, interquartile range; ND, not determined.

a Kruskal Wallis test.

b χ2.

c Data presumed due to the long-lived exposure to malaria of malaria-exposed adults in the living area.

d Mann-Whitney test.

e Parasitemia data was available in 45 children, 20 travelers, 14 expatriates, 90 malaria-exposed adults.

### IgG antibody responses during a first malaria episode

IgG antibody levels against recombinant proteins and *P. falciparum* IEs were measured in children and travelers presenting with a first malaria episode on the day of malaria diagnosis and at convalescence. Merozoite proteins AMA-1, MSP-1_42_, EBA-175 were chosen because they are involved in essential parasite functions (erythrocyte invasion) and are targets of naturally acquired antibodies associated with malaria immunity and thus are leading vaccine candidates [21]–[25]. The R29 isolate was used because it forms rosettes, a mechanism involved in severe malaria, and that predominantly expresses a group A *Pf*EMP-1 [13], to which antibodies are rapidly acquired in children [26]. DBL-α is a *Pf*EMP-1 domain cloned from the R29 lab isolate that is involved in rosetting through adhesion to complement receptor 1 (R29var1 [27]). In addition, prior pilot studies with several laboratory parasite isolates in the Manhiça area showed that R29 is the most immunogenic when measuring IgGs to variant surface antigens (VSA) (data not shown). The CS2 strain was included because it is a parasite isolate from pregnant women that binds to CSA, and an extensive and persistent recognition of VAR2CSA by plasma from children has been reported [28]. Finally, DBL3X was included because it is a VAR2CSA domain involved in CSA binding.

IgG antibody levels in plasma against recombinant proteins and IEs were not significantly different in children and travelers during a first malaria episode with the exception of MSP-1_42_ 3D7 and IE_Woman_ isolate (Table 2). Children had higher levels of IgG against MSP-1_42_ 3D7 and IE_Woman_ than travelers (*P* = 0.030 and *P* = 0.002, respectively). Similarly, there were no differences in antibody prevalences or in breadth of response (data not shown) with the exception of IgG prevalence against MSP-1_42_ 3D7 that was higher in children than travelers (*P* = 0.027) (Table 2).

**Table 2:** Plasma IgG antibody levels and seroprevalences (number and % of responders) against recombinant proteins and IEs surface antigens in children and naïve adults in the acute phase of a first *P. falciparum* malaria episode.

	IgG levels (AU)					Prevalences				
	Children n = 30		Travelers n = 20			Children n = 28		Travelers n = 20		
Antigen	Median	(IQR)	Median	(IQR)	*P-value* a	n	%	n	%	*P-value* b
Proteins c										
AMA-1 3D7	758.74	(202.22; 8402.47)	287.84	(77.22; 1471.94)	*0.075*	19	*68*	9	*45*	*0.144*
AMA-1 FVO	1036.82	(477.63; 7260.11)	823.74	(293.71; 4171.51)	*0.403*	11	*39*	6	*30*	*0.555*
MSP-1 3D7	3369.76	(1578.10; 14002.25)	1172.45	(389.85; 8370.79)	*0.030*	23	*82*	10	*50*	*0.027*
MSP-1 FVO	1094.26	(301.89; 13641.75)	745.68	(147.21; 6985.65)	*0.380*	15	*54*	8	*40*	*0.394*
EBA-175	358.73	(21.30; 802.77)	344.32	(8.54; 663.42)	*0.900*	0	*0*	0	*0*	*ND*
DBLα	493.55	(220.83; 901.79)	849.98	(220.35; 1091.30)	*0.452*	4	*14*	3	*15*	*1.000*
DBL3X	114.06	(76.11; 309.35)	133.02	(50.62; 329.63)	*0.786*	8	*29*	6	*30*	*1.000*
IEs										
IE_Trav1_	26.43	(22.87; 32.30)	25.3	(20.31; 33.22)	*0.458*	7	*23*	4	*20*	*1.000*
IE_Trav2_	30.75	(26.69; 39.98)	32.56	(26.28; 43.48)	*0.678*	8	*27*	7	*35*	*0.547*
IE_Trav3_	10.23	(8.00; 13.77)	9.23	(7.74; 11.70)	*0.294*	2	*7*	1	*5*	*1.000*
CS2	8.91	(8.09; 9.22)	9.62	(8.84; 10.46)	*0.057*	3	*10*	0	*0*	*0.265*
R29	13.65	(11.27; 15.93)	16.93	(12.81; 22.07)	*0.083*	6	*20*	7	*35*	*0.327*
IE_Ch1_	3.21	(2.06; 5.12)	2.7	(1.89; 3.13)	*0.148*	3	*10*	1	*5*	*0.641*
IE_Woman_	3.76	(3.32; 4.45)	3.16	(2.68; 3.44)	*0.002*	3	*10*	1	*5*	*0.641*
IE_Ch2_	0.73	(0.47; 1.83)	0.62	(0.13; 1.18)	*0.201*	6	*20*	2	*10*	*0.450*

Abbreviations: AU, Arbitrary units; IQR, interquartile range; ND, not determined; IEs, infected erythrocytes.

a Mann-Whitney test.

b Fisher's exact test.

c Determinations were done in children n = 29.

At convalescence, there were no significant differences in IgG levels or prevalences to recombinant proteins (Table 3). Children born from mothers with placental malaria infection did not show any significant differences in antibody responses compared to those born from mothers without placental malaria infection (data not shown).

**Table 3:** Plasma IgG levels and seroprevalences in children and travelers in convalescence (day 28) after a first episode of *P. falciparum* malaria.

	IgG levels (AU)					Prevalences				
	Children n = 28		Travelers n = 6			Children n = 28		Travelers n = 6		
Antigens	Median	(IQR)	Median	(IQR)	*P-value* a	n	%	n	%	*P-value* b
Proteins										
AMA-1 3D7	12459.59	(2442.78; 25005.45)	7211.44	(516.28; 11636.50)	*0.5571*	25	89	6	100	*1.000*
AMA-1 FVO	13248.19	(1622.11; 23681.75)	12544.35	(7112.02; 21767.80)	*0.7861*	21	75	5	83	*1.000*
MSP-1 3D7	21629.75	(17970.65; 30033.10)	21027.6	(16816.20; 22336.90)	*0.4981*	27	96	6	100	*1.000*
MSP-1 FVO	24619.3	(17512.80; 33060.50)	26869.15	(20308.50; 30278.10)	*0.6191*	25	89	6	100	*1.000*
EBA-175	595.58	(38.64; 1251.93)	753.07	(290.80; 1070.08)	*0.7181*	3	11	6	100	*1.000*
DBLα	410.65	(313.82; 993.56)	821.74	(271.98; 1430.16)	*0.4421*	3	11	5	83	*1.000*
DBL3X	137.11	(82.10; 656.53)	94.51	(74.86; 131.44)	*0.3201*	9	32	0	0	*0.559*

Abbreviations: AU, Arbitrary units; IQR, interquartile range.

a Mann-Whitney test.

b Fisher's exact test.

### Cytokine and chemokine responses during a first malaria episode

**Figure 1:**
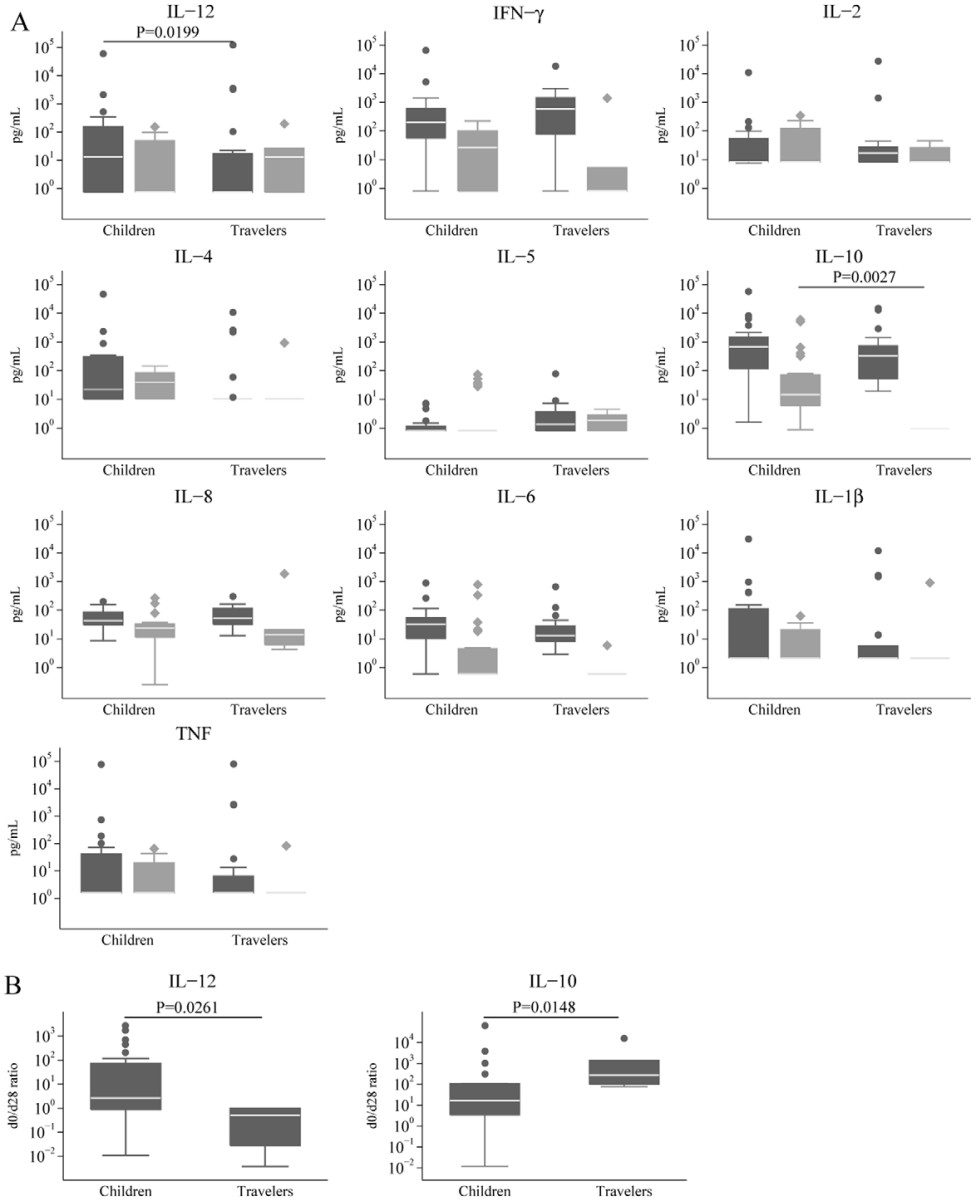
Plasma/serum cytokine levels in children and adults during a first malaria episode. A) Plasma/serum cytokine/chemokine concentrations in the acute phase (dark grey) and convalescence (light grey) were compared between children and adults using the Mann-Whitney test. B) Significantly different day 0/day 28 ratio cytokine levels in children and adults.

Cytokine and chemokine concentrations in plasma or serum were measured in children and travelers with a first malaria acute episode and during convalescence. No differences were detected in any of the cytokines/chemokines tested with the exception of IL-12 (Figure 1A). In the acute phase, children had significantly higher levels of IL-12 than travelers (median {IQR} of 13.33 {0.75; 191.81} pg/mL and 0.75 {0.75; 17.98} pg/mL, respectively; *P = *0.020). During convalescence children had significantly higher levels of IL-10 (14.66 {6.09; 70.81} pg/mL) compared to travelers (0.95 {0.95; 0.95} pg/mL, *P = *0.003). The day 0/day 28 ratio was significantly higher for IL-12 in children compared to travelers (2.66 {0.89; 72.89} and 0.52 {0.03; 1.00}, respectively; *P = *0.026) and significantly lower for IL-10 (16.41 {.46; 110.16} and 277.33 {100.77; 1406.74}, respectively; *P = *0.015) (Figure 1B).

IL-10 levels correlated moderately with parasite density in both groups whereas IL-8, IL-6 and IL-1β concentrations correlated only in travelers (Table S2). There were no correlations between cytokine and antibody levels (data not shown). In children, no association was found between cytokine levels and being born from a mother with placental malaria (data not shown).

### IgG antibody responses in differently malaria-exposed individuals

To study how exposure to malaria is affecting the IgG antibody responses to different antigens, we compared IgG levels (Table 4) and prevalences (Table S1) in children, travelers and expatriates versus life-long malaria-exposed adults. Individuals with a primo-infection (children and travelers) had significantly lower levels of IgG than malaria-exposed adults (*P<*0.001*)* regardless of the antigen. Expatriates with previous few exposures to malaria still had significantly lower levels of antibodies compared to life-long malaria-exposed individuals although the levels were higher than naïve subjects with the exceptions of IgG to MSP-1_42_ FVO, EBA-175 and DBL-α. Breadth of response was also significantly lower in primo-infected individuals or expatriates compared to malaria-exposed adults (data not shown).

**Table 4:** Plasma IgG levels in an acute episode of differently exposed individuals.

	IgG levels, median AU (IQR)				*P-value* a		
Antigens	Children n = 30	Travelers n = 20	Expatriates n = 14	Malaria-exposed n = 50	*Child-* *MalExp*	*Trav-* *MalExp*	*Expat-* *MalExp*
Proteins b							
AMA-1 3D7	758.74 (202.22; 8402.47)	287.84 (77.22; 1471.94)	12128 (565.27; 22844.40)	24455.2 (19811.30; 27728.20)	*<0.001*	*<0.001*	*0.001*
AMA-1 FVO	1036.82 (477.63; 7260.11)	823.74 (293.71; 4171.51)	16090 (154.64; 25124.90)	23957.35 (19613.80; 26059.20)	*<0.001*	*<0.001*	*0.020*
MSP-1 3D7	3369.76 (1578.10; 14002.25)	1172.45 (389.85; 8370.79)	24565.2 (12456.30; 29953.00)	21651.4 (15681.70; 23614.60)	*<0.001*	*<0.001*	*0.057*
MSP-1 FVO	1094.26 (301.89; 13641.75)	745.68 (147.21; 6985.65)	25396.85 (10079.10; 28122.50)	22546.95( 9320.43; 24863.10)	*<0.001*	*<0.001*	*0.242*
EBA-175	358.73 (21.30; 802.77)	344.32 (8.54; 663.42)	1220.22 (776.30; 6980.53)	4214.73 (837.81; 22594.80)	*<0.001*	*<0.001*	*0.284*
DBLα	493.55 (220.83; 901.79)	849.98 (220.35; 1091.30)	665.61 (409.81; 1980.22)	1353.96 (789.37; 2606.57)	*<0.001*	*0.019*	*0.163*
DBL3X	114.06 (76.11; 309.35)	133.02 (50.62; 329.63)	113.99 (52.13; 281.91)	306.06 (210.63; 1042.82)	*<0.001*	*0.002*	*0.004*
IEs c							
IE_Trav1_	26.43 (22.87; 32.30)	25.3 (20.31; 33.22)	33.62 (21.40; 40.94)	207.26 (121.95; 396.35)	*<0.001*	*<0.001*	*<0.001*
IE_Trav2_	30.75 (26.69; 39.98)	32.56 (26.28; 43.48)	36.86 (26.47; 44.68)	251.63 (135.58; 437.20)	*<0.001*	*<0.001*	*<0.001*
IE_Trav3_	10.23 (8.00; 13.77)	9.23 (7.74; 11.70)	19.74 (7.04; 21.27)	169.19 (71.57; 286.32)	*<0.001*	*<0.001*	*<0.001*
CS2	8.91 (8.09; 9.22)	9.62 (8.84; 10.46)	9.63 (8.65; 10.16)	14.03 (11.63; 27.38)	*<0.001*	*<0.001*	*0.006*
R29	13.65 (11.27; 15.93)	16.93 (12.81; 22.07)	15.36 (15.08; 35.63)	214.29 (87.23; 393.92)	*<0.001*	*<0.001*	*0.001*
IE_Ch1_	3.21 (2.06; 5.12)	2.7 (1.89; 3.13)	3.85 (3.39; 7.44)	63.28 (30.99; 123.09)	*<0.001*	*<0.001*	*<0.001*
IE_Woman_	3.76 (3.32; 4.45)	3.16 (2.68; 3.44)	3.73 (2.82; 5.15)	21.43 (11.92; 46.10)	*<0.001*	*<0.001*	*0.001*
IE_Ch2_	0.73 (0.47; 1.83)	0.62 (0.13; 1.18)	1.34 (−0.14; 1.49)	41.69 (22.40; 81.08)	*<0.001*	*<0.001*	*<0.001*

Abbreviations: AU, Arbitrary units; IQR, Interquartile range; MalExp, Malaria-Exposed; Trav, Travelers; Expat, Expatriates; IEs, Infected erythrocytes.

a The Mann-Whitney test was used.

b Determinations were done in children n = 28.

c Determinations were done in expatriates n = 5.

### Cytokine and chemokine responses in differently malaria-exposed individuals

**Figure 2:**
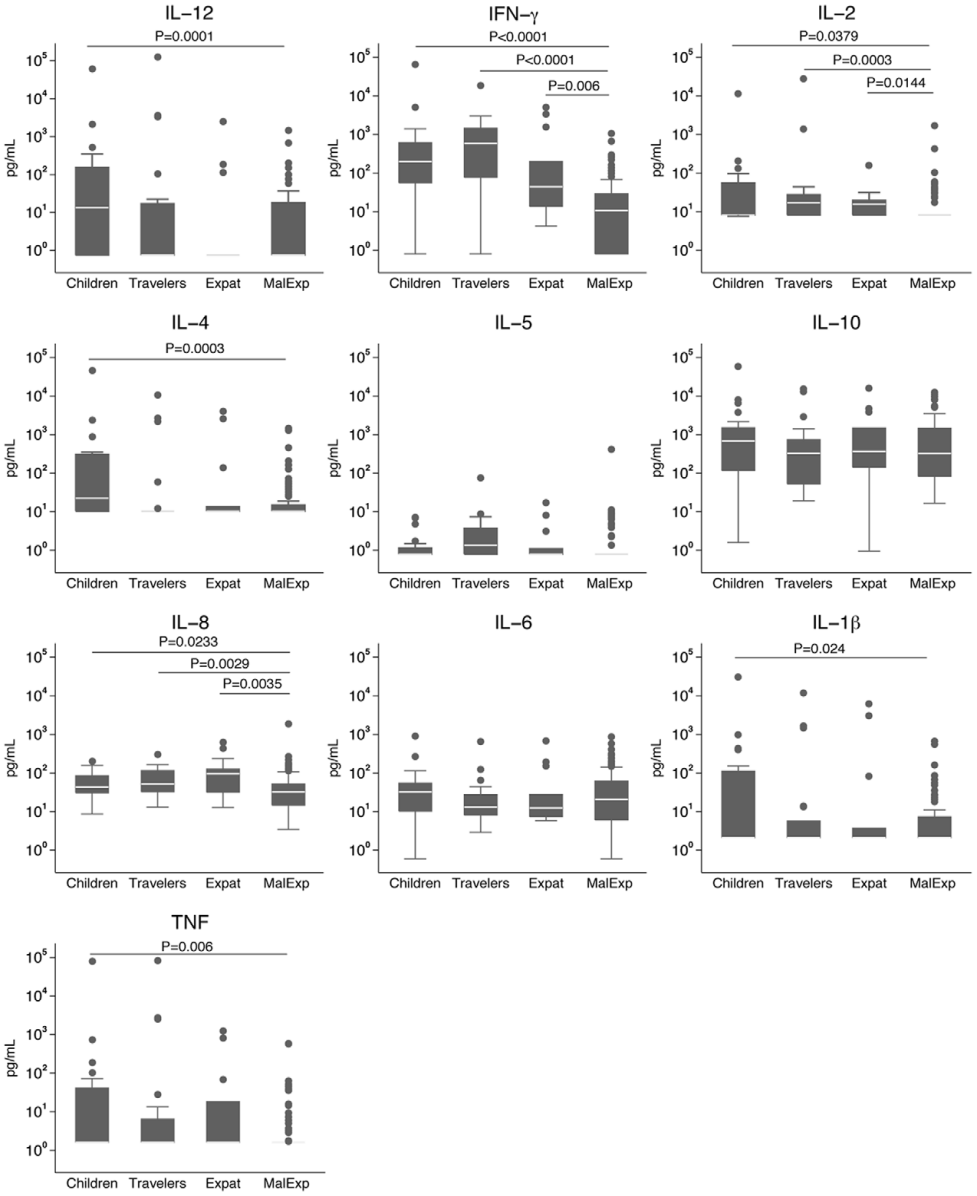
Plasma/serum cytokine/chemokine concentrations (pg/mL) in the acute phase of differently exposed subjects. Mann-Whitney test was used to compare children, travelers and expatriates (Expat) to malaria-exposed adults (MalExp).

Cytokine measurements in individuals with an acute episode showed that children, travelers and expatriates had significantly higher levels of IFN-γ than malaria-exposed individuals (median {IQR} of 163.85 {51.33; 564.16}, 584.54 {77.17; 1446.56}, 44.03 {13.7; 199.72}, and 10.65 {0.8; 29} pg/mL, respectively; *P<*0.0001, *P<*0.0001, *P = *0.006, respectively) (Figure 2). In addition, significantly higher levels of IL-2 were detected in children (8.2 {8.2; 54.93} pg/mL), travelers (17.03 {8.2; 27.42} pg/mL) and expatriates (15.77 {8.2; 19.7} pg/mL) compared to malaria-exposed adults (8.2 {8.2; 8.2} pg/mL; *P = 0.0379, P = *0.0003, *P = *0.0144, respectively). Children, travelers and expatriates also had significantly higher concentrations of IL-8 (41.97 {30.02; 55.7}, 52.2 {32.72; 114.69} and 95.31 {31.86; 126.26} pg/mL, respectively) in comparison with malaria-exposed adults (32.2 {14.69; 51.61}; *P = *0.0233, *P = *0.0029, *P = *0.0035, respectively). Compared to malaria-exposed adults, children also had higher levels of IL-12 (13.33 {0.75; 191.81} vs. 0.75 {0.75; 17.98} pg/mL; *P = *0.0001), IL-4 (10.94 {10.4; 333.6} vs. 10.4 {10.4; 14.99} pg/mL; *P = *0.003), IL-1β (2.1 {2.1; 132.18} vs. 2.1{2.1; 7.3} pg/mL; *P = *0.024) and TNF (1.6 {1.6; 56.97} vs. 1.6 {1.6; 1.6} pg/mL; *P = *0.006).

In addition to the correlations between cytokines and parasitemia in children and travelers described above, IFN-γ, IL-4 and IL-1β correlated significantly with parasitemia only in the expatriates group (Table S2). None of the cytokines/chemokines tested correlated significantly with parasitemia in malaria-exposed adults.

No correlations between cytokines and antibodies were observed in any study group (data not shown).

## Discussion

Results comparing naïve adults and children indicate that age has no prominent role in the immune responses induced during a first malaria episode. Differences were found only in IgG responses to MSP-1_42_ 3D7 and to a parasite from a pregnant woman, and in IL-12 concentrations in serum/plasma. All three responses were higher in children. In addition, at convalescence children had higher levels of IL-10 and there were no differences in antibody levels or seroprevalences, although for travelers only 6 samples out of 22 were available at day 28 of convalescence.

IL-12 has been associated with protection against malaria [29], [30] and severe disease [31], [32]. IL-12 is a T_H_1 cytokine produced mainly by dendritic cells (DC) in response to Toll-Like-Receptor (TLR) stimulation. It induces and regulates DC maturation and function and promotes IFN-γ production by T cells and NK cells as well as their activation [33], [34]. Here increased levels of IL-12 indicate a higher innate immune response in younger children, probably due to an age-differential response to TLR ligands [4]. This could contribute to the lower risk of severe complications and different clinical presentations described in naïve adults compared to children [2], [12].

At convalescence, children had higher levels of the anti-inflammatory cytokine IL-10 than naïve adults. Therefore, the total change in magnitude of this cytokine in acute vs. convalescence was significantly lower in children compared to adults, suggesting different capacity of regulating the pro-inflammatory response. The observation that children had higher levels of TNF and IL-1β in acute phase compared to life-long malaria-exposed adults also suggests a different ability to regulate pro-inflammatory and anti-inflammatory responses according to age and/or exposure. Nevertheless, most children were *P. falciparum* PCR positive at day 28 (data not shown), although they were treated with anti-malarial drugs and they did not have fever or microscopic parasitemia. Conversely, none of the adults were PCR positive at convalescence. It is possible that the higher IL-10 levels at convalescence were reflecting a response to submicroscopic parasitemia from uncleared/recrudescent or new infections. However, the most plausible explanation is that PCR positives were due to residual parasite DNA in dead IEs remaining in circulation [35].

Comparisons between individuals with different levels of exposure to *P. falciparum* allowed us to identify IFN-γ, IL-2 and IL-8 as cytokines/chemokines involved in cellular immune responses to *P. falciparum*. As expected, increasing exposure showed to be associated to an increase of antibody levels against any antigen, but in this study we were unable to evaluate its role in immunity. Compared to antibodies, the more moderate cytokine responses may reflect naturally acquired immunity that allows a better control of pro-inflammatory responses rather than mere exposure. Although IFN-γ has been associated with malaria protection [29], [36] and IL-2 may be key for the generation of effector responses to malaria [36], [37], the strong T_H_1 and inflammatory responses detected reflect innate rather than protective adaptive immune responses. Moreover, levels of IL-1β and TNF were higher in children compared to malaria-exposed adults during acute infection despite having a trend to lower parasite density than malaria-exposed adults mounting a higher antibody response to infection. This reinforces the idea that naïve individuals have stronger pro-inflammatory responses that would be independent from the parasite biomass and may be key factors to the prognosis of clinical episodes. Children also presented significantly higher IL-4 levels compared to malaria-exposed adults. It has been described that IL-4 promotes IL-12 DC secretion [38], which is in line with the higher IL-12 levels found in children compared to adults. The higher T_H_1/pro-inflammatory responses observed in naïve individuals were not caused by higher parasite densities. Although we could not compare parasitemias between all groups because of different methods used to assess parasite density, there were no significant differences between children and malaria-exposed adults or between travelers and expatriates. Additionally, correlations between cytokines and parasitemia showed that cytokines differently expressed in naïve versus malaria-exposed individuals (IL-12, IFN-γ, IL-2, IL-1β and TNF) did not show to be associated with parasitemia with the exception of IL-8 in naïve adults. These results suggest that these cytokines are not directly affected by parasitemia and the differences observed in cytokine concentrations may not be a direct effect of differences in parasite densities among the groups. Moreover, the fact that the correlations between cytokines and parasite densities were different among groups might be a reflection of different mechanisms of cytokine regulation depending on age and immunity.

Hence, our data may be reflecting development of tolerance, a recognized mechanism of acquisition of immunity to *P. falciparum* [39]. Tolerance has been described to be associated with less expression of co-stimulatory molecules, less IL-6, IL-12, TNF and IFN-γ (pro-inflammatory cytokines) and an attenuation of cell signaling. Mechanisms may include TLR tolerance due to repeated antigen stimulation [40], [41], tolerogenic DC [42], [43], regulatory T cells [44], [45] and exhausted T cells [46].

Antibody seroprevalences in children and naïve adults were quite elevated with the exception of EBA-175, considering that IgG levels were determined at the acute phase of a first malaria episode. Study children were from a malaria endemic area and it is plausible that they might have been previously exposed to *P. falciparum* infection (i.e. after birth or in utero [47]). However, they were followed up closely by active and passive morbidity surveillance and we are quite confident that we captured the first malaria episode. In addition, cytokine and antibody responses did not show any significant association with in utero exposure through maternal malaria infection (placental infection or cord infection). In fact, IgG responses to most antigens, except those described above, were not higher in children than adults. Regarding IgG responses to the pregnant woman parasite isolate, it could be due to passively transferred maternal antibodies against CSA-binding parasites, however it is unlikely that these children still had antibodies from the mother because they had a median age of 1.1 years with the youngest one being 10.8 months old. IgG detected in seropositive children and travelers are probably reflecting the beginning of the antibody response to blood-stage antigens even if few days had passed since the release of merozoites from the liver [48]. Antibody responses were quite low and both IgG levels and seroprevalences increased considerably at convalescence, and were consistently lower than those of expatriates or life-long malaria-exposed adults. In addition, several days may have passed before traveler patients attended the Tropical Medicine Unit.

Overall, antibody results are consistent with previous data of acquired immunity in which there are two levels of immunity, one strain-specific that is slowly acquired by consecutive exposures to polymorphic antigens and another transcending antigenic diversity [8]. In travelers, a single *P. falciparum* infection can be sufficient to induce antibodies reactive with several *Pf*EMP-1 variants that are already detected at initial presentation to the hospital [48]. Also a quite extensive breath of agglutination specificities was reported in migrants from a non-endemic to an endemic zone considering a relatively short time of exposure [49]. However, the degree of cross-reactivity among strains seems to correlate with age [8]. In our study, no differences in median breadth of response were detected between children and naïve adults against recombinant proteins or VSAs, which had a very low breadth response. In contrast, expatriates were responding to more antigens and malaria-exposed adults showed a significantly higher breadth of response compared to less exposed groups. The fact that travelers were exposed to geographically distinct strains of *P. falciparum* seems not to affect the antibody response to the tested antigens and VSAs from Mozambican strains as they responded similarly to children from Mozambique. Similar observations were reported in people with limited histories of exposure who showed broad agglutination activity against geographically diverse isolates [50] or in a study were IgG VSA recognition was independent of the geographical origin of parasites [51].

IgG seroprevalence to DBL-α in children was about 15%, lower than what has been described in Manhiça children [32], although in that study children were older and some have had previous malaria episodes. Antibodies against DBL-α have been linked to natural acquisition of immunity [26], to reduced risk of clinical malaria [52], and have been associated with protection from severe malaria [32]. The prevalence of antibodies against DBL3X was about 30% in children, which is similar to a recent study that suggested recognition of the VAR2CSA DBL5ε domain in young children [28]. Therefore our results contribute to the hypothesis that exposure to VAR2CSA-expressing parasites is probably common throughout early childhood [53]. Nevertheless, the recognition of pregnancy-specific CSA-binding parasites was about 10% in children and less in travelers.

To our knowledge only antibodies against MSP-2 3D7, RESA and GPI have been correlated with age independently of exposure [49], [54]. Our study is limited by a number of factors that may mask the detection of other age-dependent differences. For instance, differences due to the African vs. European origin of the comparison groups may confound the results. IgM, IgG isotype levels and antibody affinity need to be determined in follow up studies. IgM responses are relevant in a first malaria episode and the protective effect of IgG isotypes has been attributed to the cytophilic subclasses IgG1 and IgG3 [22], [55], [56]. In addition, affinity seems to increase with age and is associated with protection [57].

In conclusion, age appeared not to be a significant factor in the immune response to a first malaria episode, particularly for the antibody responses examined. However, some observations such as higher levels of IL-12 or higher pro-inflammatory responses in children may indicate that some cellular age-dependent responses have remained undetected in this study. Exposure to *P. falciparum* was clearly associated with higher levels of IgG antibodies against merozoite antigens and IEs, although we could not dissect their role in immunity. Exposure to malaria and consequently, naturally acquired immunity, was associated with lower levels of T_H_1 and pro-inflammatory cytokines, indicating a more regulated and balanced innate response related to mechanisms of tolerance.

## Supporting Information

Table S1 **Seroprevalence (number and % of responders) of IgG antibody responses in a malaria acute episode against recombinant proteins and IEs surface antigens in differently exposed individuals.**
(PDF)

Table S2 **Spearman correlations between cytokines/chemokines and parasitemia.**
(PDF)

## References

[1] WHO. World Malaria Report 2011 (2011)

[2] Doolan DL, Dobaño C, Baird JK (2009) Acquired immunity to malaria. Clin Microbiol Rev 22: 13–36. 19136431 10.1128/CMR.00025-08 PMC2620631

[3] Prabhudas M, Adkins B, Gans H, King C, Levy O, et al (2011) Challenges in infant immunity: implications for responses to infection and vaccines. Nat Immunol 12: 189–194. 21321588 10.1038/ni0311-189

[4] Corbett NP, Blimkie D, Ho KC, Cai B, Sutherland DP, et al (2010) Ontogeny of Toll-like receptor mediated cytokine responses of human blood mononuclear cells. PloS One 5: e15041. 21152080 10.1371/journal.pone.0015041 PMC2994830

[5] Guinovart C, Dobaño C, Bassat Q, Nhabomba A, Quintó L, et al (2012) The Role of Age and Exposure to Plasmodium falciparum in the Rate of Acquisition of Naturally Acquired Immunity: A Randomized Controlled Trial. PloS One 7: e32362. 22412865 10.1371/journal.pone.0032362 PMC3296698

[6] Aponte JJ, Menendez C, Schellenberg D, Kahigwa E, Mshinda H, et al (2007) Age interactions in the development of naturally acquired immunity to Plasmodium falciparum and its clinical presentation. PLoS Med 4: e242. 17676985 10.1371/journal.pmed.0040242 PMC1950208

[7] Baird JK, Jones TR, Danudirgo EW, Annis BA, Bangs MJ, et al (1991) Age-dependent acquired protection against Plasmodium falciparum in people having two years exposure to hyperendemic malaria. Am J Trop Med Hyg 45: 65–76. 1867349 10.4269/ajtmh.1991.45.65

[8] Baird JK (1995) Host age as a determinant of naturally acquired immunity to Plasmodium falciparum. Parasitol Today 11: 105–111. 15275362 10.1016/0169-4758(95)80167-7

[9] Bouchaud O, Cot M, Kony S, Durand R, Schiemann R, et al (2005) Do African immigrants living in France have long-term malarial immunity? Am J Trop Med Hyg 72: 21–25. 15728861

[10] Salvadó E, Pinazo MJ, Muñoz J, Alonso D, Naniche D, et al (2008) Clinical presentation and complications of Plasmodium falciparum malaria in two populations: travelers and immigrants. Enferm Infecc Microbiol Clin 26: 282–284. 18479645 10.1157/13120415

[11] Baird JK, Masbar S, Basri H, Tirtokusumo S, Subianto B, et al (1998) Age-dependent susceptibility to severe disease with primary exposure to Plasmodium falciparum. J Infect Dis 178: 592–595. 9697752 10.1086/517482

[12] Marsh K, Snow RW (1999) Malaria transmission and morbidity. Parassitologia 41: 241–246. 10697862

[13] Rovira-Vallbona E, Dobaño C, Bardají A, Cisteró P, Romagosa C, et al (2011) Transcription of var genes other than var2csa in Plasmodium falciparum parasites infecting Mozambican pregnant women. J Infect Dis 204: 27–35. 21628655 10.1093/infdis/jir217 PMC3307158

[14] Kocken CHM, Withers-Martinez C, Dubbeld MA, Van der Wel A, Hackett F, et al (2002) High-level expression of the malaria blood-stage vaccine candidate Plasmodium falciparum apical membrane antigen 1 and induction of antibodies that inhibit erythrocyte invasion. Infect Immun 70: 4471–4476. 12117958 10.1128/IAI.70.8.4471-4476.2002 PMC128198

[15] Pandey KC, Singh S, Pattnaik P, Pillai CR, Pillai U, et al (2002) Bacterially expressed and refolded receptor binding domain of Plasmodium falciparum EBA-175 elicits invasion inhibitory antibodies. Mol Biochem Parasitol 123: 23–33. 12165386 10.1016/s0166-6851(02)00122-6

[16] Mayor A, Rovira-Vallbona E, Srivastava A, Sharma SK, Pati SS, et al (2009) Functional and immunological characterization of a Duffy binding-like alpha domain from Plasmodium falciparum erythrocyte membrane protein 1 that mediates rosetting. Infect Immun 77: 3857–3863. 19546191 10.1128/IAI.00049-09 PMC2738012

[17] Bir N, Yazdani SS, Avril M, Layez C, Gysin J, et al (2006) Immunogenicity of Duffy binding-like domains that bind chondroitin sulfate A and protection against pregnancy-associated malaria. Infect Immun 74: 5955–5963. 16988275 10.1128/IAI.00481-06 PMC1594931

[18] Angov E (2003) Development and pre-clinical analysis of a Plasmodium falciparum Merozoite Surface Protein-142 malaria vaccine. Mol Biochem Parasitol 128: 195–204. 12742586 10.1016/s0166-6851(03)00077-x

[19] Angov E, Hillier CJ, Kincaid RL, Lyon JA (2008) Heterologous protein expression is enhanced by harmonizing the codon usage frequencies of the target gene with those of the expression host. PloS One 3: e2189. 18478103 10.1371/journal.pone.0002189 PMC2364656

[20] Campo JJ, Dobaño C, Sacarlal J, Guinovart C, Mayor A, et al (2011) Impact of the RTS,S Malaria Vaccine Candidate on Naturally Acquired Antibody Responses to Multiple Asexual Blood Stage Antigens. PloS One 6: e25779. 22022448 10.1371/journal.pone.0025779 PMC3192128

[21] Richards JS, Stanisic DI, Fowkes FJI, Tavul L, Dabod E, et al (2010) Association between naturally acquired antibodies to erythrocyte-binding antigens of Plasmodium falciparum and protection from malaria and high-density parasitemia. Clin Infect Dis 51: e50–60. 20843207 10.1086/656413

[22] Dobaño C, Quelhas D, Quintó L, Puyol L, Serra-Casas E, et al (2012) Age-dependent IgG subclass responses to Plasmodium falciparum EBA-175 are differentially associated with incidence of malaria in Mozambican children. Clin Vaccine Immunol 19: 157–166. 22169088 10.1128/CVI.05523-11 PMC3272921

[23] Dodoo D, Aikins A, Kusi KA, Lamptey H, Remarque E, et al (2008) Cohort study of the association of antibody levels to AMA1, MSP119, MSP3 and GLURP with protection from clinical malaria in Ghanaian children. Malar J 7: 142. 18664257 10.1186/1475-2875-7-142 PMC2529305

[24] Conway DJ, Cavanagh DR, Tanabe K, Roper C, Mikes ZS, et al (2000) A principal target of human immunity to malaria identified by molecular population genetic and immunological analyses. Nat Med 6: 689–692. 10835687 10.1038/76272

[25] Polley SD, Mwangi T, Kocken CHM, Thomas AW, Dutta S, et al (2004) Human antibodies to recombinant protein constructs of Plasmodium falciparum Apical Membrane Antigen 1 (AMA1) and their associations with protection from malaria. Vaccine 23: 718–728. 15542195 10.1016/j.vaccine.2004.05.031

[26] Cham GKK, Turner L, Lusingu J, Vestergaard L, Mmbando BP, et al (2009) Sequential, ordered acquisition of antibodies to Plasmodium falciparum erythrocyte membrane protein 1 domains. J Immunol 183: 3356–3363. 19675168 10.4049/jimmunol.0901331

[27] Mayor A, Rovira-Vallbona E, Machevo S, Bassat Q, Aguilar R, et al (2011) Parity and Placental Infection Affect Antibody Responses against Plasmodium falciparum during Pregnancy. Infect Immun 79: 1654–1659. 21300778 10.1128/IAI.01000-10 PMC3067565

[28] Oleinikov A V, Voronkova V V, Frye IT, Amos E, Morrison R, et al (2012) A plasma survey using 38 PfEMP1 domains reveals frequent recognition of the Plasmodium falciparum antigen VAR2CSA among young Tanzanian children. PLoS ONE 7: e31011. 22295123 10.1371/journal.pone.0031011 PMC3266279

[29] Sedegah M, Finkelman F, Hoffman SL (1994) Interleukin 12 induction of interferon gamma-dependent protection against malaria. Proc Natl Acad Sci U S A 91: 10700–10702. 7938013 10.1073/pnas.91.22.10700 PMC45089

[30] Hoffman SL, Crutcher JM, Puri SK, Ansari AA, Villinger F, et al (1997) Sterile protection of monkeys against malaria after administration of interleukin-12. Nat Med 3: 80–83. 8986746 10.1038/nm0197-80

[31] Perkins DJ, Weinberg JB, Kremsner PG (2000) Reduced interleukin-12 and transforming growth factor-beta1 in severe childhood malaria: relationship of cytokine balance with disease severity. J Infect Dis 182: 988–992. 10950804 10.1086/315762

[32] Rovira-Vallbona E, Moncunill G, Bassat Q, Aguilar R, Machevo S, et al (2012) Low antibodies against Plasmodium falciparum and imbalanced pro-inflammatory cytokines are associated with severe malaria in Mozambican children: a case-control study. Malar J 11: 181. 22646809 10.1186/1475-2875-11-181 PMC3464173

[33] Langrish CL, McKenzie BS, Wilson NJ, De Waal Malefyt R, Kastelein R a, et al (2004) IL-12 and IL-23: master regulators of innate and adaptive immunity. Immunol Rev 202: 96–105. 15546388 10.1111/j.0105-2896.2004.00214.x

[34] Torre D (2009) Early production of gamma-interferon in clinical malaria: role of interleukin-18 and interleukin-12. Clin Infect Dis 48: 1481–1482. 19374561 10.1086/598508

[35] Mayor A, Moro L, Aguilar R, Bardají A, Cisteró P, et al (2012) How hidden can malaria be in pregnant women? Diagnosis by microscopy, placental histology, polymerase chain reaction and detection of histidine-rich protein 2 in plasma. Clin Infect Dis 54: 1561–1568. 22447794 10.1093/cid/cis236

[36] McCall MBB, Sauerwein RW (2010) Interferon-γ – central mediator of protective immune responses against the pre-erythrocytic and blood stage of malaria. J Leukoc Biol 88: 1131–1143. 20610802 10.1189/jlb.0310137

[37] Horowitz A, Newman KC, Evans JH, Korbel DS, Davis DM, et al (2010) Cross-talk between T cells and NK cells generates rapid effector responses to Plasmodium falciparum-infected erythrocytes. J Immunol 184: 6043–6052. 20427769 10.4049/jimmunol.1000106

[38] Cook PC, Jones LH, Jenkins SJ, Wynn T a, Allen JE, et al (2012) Alternatively activated dendritic cells regulate CD4+ T-cell polarization in vitro and in vivo. Proc Natl Acad Sci U S A 109: 2–7. 10.1073/pnas.1121231109 PMC3382483 22660926

[39] Boutlis CS, Yeo TW, Anstey NM (2006) Malaria tolerance – for whom the cell tolls? Trends Parasitol 22: 371–377. 16784889 10.1016/j.pt.2006.06.002 PMC2766419

[40] Perry JA, Olver CS, Burnett RC, Avery AC (2005) Cutting edge: the acquisition of TLR tolerance during malaria infection impacts T cell activation. J Immunol 174: 5921–5925. 15879082 10.4049/jimmunol.174.10.5921

[41] Broad A, Kirby JA, Jones DEJ (2007) Toll-like receptor interactions: tolerance of MyD88-dependent cytokines but enhancement of MyD88-independent interferon-beta production. Immunology 120: 103–111. 17034424 10.1111/j.1365-2567.2006.02485.x PMC2265871

[42] Rutella S, Danese S, Leone G (2006) Tolerogenic dendritic cells: cytokine modulation comes of age. Blood 108: 1435–1440. 16684955 10.1182/blood-2006-03-006403

[43] Stevenson MM, Urban BC (2006) Antigen presentation and dendritic cell biology in malaria. Parasite Immunol 28: 5–14. 16438671 10.1111/j.1365-3024.2006.00772.x

[44] Hansen DS, Schofield L (2010) Natural regulatory T cells in malaria: host or parasite allies? PLoS Pathog 6: e1000771. 20442856 10.1371/journal.ppat.1000771 PMC2861684

[45] Walther M, Jeffries D, Finney OC, Njie M, Ebonyi A, et al (2009) Distinct roles for FOXP3 and FOXP3 CD4 T cells in regulating cellular immunity to uncomplicated and severe Plasmodium falciparum malaria. PLoS Pathog 5: e1000364. 19343213 10.1371/journal.ppat.1000364 PMC2658808

[46] Gigley JP, Bhadra R, Moretto MM, Khan IA (2012) T cell exhaustion in protozoan disease. Trends Parasitol 28: 377–384. 22832368 10.1016/j.pt.2012.07.001 PMC3768288

[47] Metenou S, Suguitan AL, Long C, Leke RGF, Taylor DW (2007) Fetal immune responses to Plasmodium falciparum antigens in a malaria-endemic region of Cameroon. J Immunol 178: 2770–2777. 17312120 10.4049/jimmunol.178.5.2770

[48] Elliott SR, Payne PD, Duffy MF, Byrne TJ, Tham W-H, et al (2007) Antibody recognition of heterologous variant surface antigens after a single Plasmodium falciparum infection in previously naive adults. Am J Trop Med Hyg 76: 860–864. 17488905

[49] Reeder JC, Davern KM, Baird JK, Rogerson SJ, Brown G V (1997) The age-specific prevalence of Plasmodium falciparum in migrants to Irian Jaya is not attributable to agglutinating antibody repertoire. Acta Trop 65: 163–173. 9177578 10.1016/s0001-706x(97)00661-x

[50] Aguiar JC, Albrecht GR, Cegielski P, Greenwood BM, Jensen JB, et al (1992) Agglutination of Plasmodium falciparum-infected erythrocytes from east and west African isolates by human sera from distant geographic regions. Am J Trop Med Hyg 47: 621–632. 1449203 10.4269/ajtmh.1992.47.621

[51] Nielsen MA, Vestergaard LS, Lusingu J, Kurtzhals JAL, Giha HA, et al (2004) Geographical and temporal conservation of antibody recognition of Plasmodium falciparum variant surface antigens. Infect Immun 72: 3531–3535. 15155661 10.1128/IAI.72.6.3531-3535.2004 PMC415673

[52] Mackintosh CL, Christodoulou Z, Mwangi TW, Kortok M, Pinches R, et al (2008) Acquisition of naturally occurring antibody responses to recombinant protein domains of Plasmodium falciparum erythrocyte membrane protein 1. Malar J 7: 155. 18706102 10.1186/1475-2875-7-155 PMC2533674

[53] Beeson JG, Ndungu F, Persson KEM, Chesson JM, Kelly GL, et al (2007) Antibodies among men and children to placental-binding Plasmodium falciparum-infected erythrocytes that express var2csa. Am J Trop Med Hyg 77: 22–28. 17620626

[54] Hudson Keenihan SN, Ratiwayanto S, Soebianto S, Krisin, Marwoto H, et al (2003) Age-dependent impairment of IgG responses to glycosylphosphatidylinositol with equal exposure to Plasmodium falciparum among Javanese migrants to Papua, Indonesia. Am J Trop Med Hyg 69: 36–41. 12932094

[55] Roussilhon C, Oeuvray C, Müller-Graf C, Tall A, Rogier C, et al (2007) Long-term clinical protection from falciparum malaria is strongly associated with IgG3 antibodies to merozoite surface protein 3. PLoS Med 4: e320. 18001147 10.1371/journal.pmed.0040320 PMC2071934

[56] Stanisic DI, Richards JS, McCallum FJ, Michon P, King CL, et al (2009) Immunoglobulin G subclass-specific responses against Plasmodium falciparum merozoite antigens are associated with control of parasitemia and protection from symptomatic illness. Infect Immun 77: 1165–1174. 19139189 10.1128/IAI.01129-08 PMC2643653

[57] Reddy SB, Anders RF, Beeson JG, Färnert A, Kironde F, et al (2012) High affinity antibodies to Plasmodium falciparum merozoite antigens are associated with protection from malaria. PloS ONE 7: e32242. 22363818 10.1371/journal.pone.0032242 PMC3283742

