# Supplementary material for: Cytokine and Antibody Responses to Plasmodium falciparum in Naïve Individuals during a First Malaria Episode: Effect of Age and Malaria Exposure
Source: PLoS One. 2013 Feb 21;8(2):e55756. doi: 10.1371/journal.pone.0055756 (PMC3578867; doi:10.1371/journal.pone.0055756)
Supplement: Table S1 — Seroprevalence (number and % of responders) of IgG antibody responses in a malaria acute episode against recombinant proteins and IEs surface antigens in differently exposed individuals. (PDF) [file pone.0055756.s001.pdf]

Table S1. Seroprevalence (number and % of responders) of IgG antibody responses in a malaria acute episode against recombinant proteins and IEs surface antigens in differently exposed individuals

	Children n=30		Travelers n=20		Expatriates n=14		Malaria-exposed n=50		*P-value* [a]		
**Antigens**	**n**	**%**	**n**	**%**	**n**	**%**	**n**	**%**	***Child-MalExp***	***Trav-MalExp***	***Expat-MalExp***
**Proteins** [b]											
AMA-1 3D7	19	68	9	45	11	79	48	96	0.001	<0.001	0.065
AMA-1 FVO	11	39	6	30	10	71	47	94	<0.001	<0.001	0.036
MSP-1 3D7	23	82	10	50	12	86	47	94	0.127	<0.001	0.299
MSP-1 FVO	15	54	8	40	13	93	46	92	<0.001	<0.001	1.000
EBA-175	0	0	0	0	5	36	25	50	<0.001	<0.001	0.381
DBLα	4	14	3	15	4	29	14	28	0.263	0.359	1.000
DBL3X	8	29	6	30	4	29	32	64	0.004	0.016	0.031
**IEs** [c]											
IE_{Trav1}	7	23	4	20	2	40	49	98	<0.001	<0.001	0.001
IE_{Trav2}	8	27	7	35	2	40	50	100	<0.001	<0.001	<0.001
IE_{Trav3}	2	7	1	5	0	0	49	98	<0.001	<0.001	<0.001

CS2	3	10	0	0	0	0	22	44	<0.001	<0.001	0.076
R29	6	20	7	35	2	40	48	96	<0.001	<0.001	0.004
IE_{Ch1}	3	10	1	5	1	20	50	100	<0.001	<0.001	<0.001
IE_{Woman}	3	10	1	5	1	20	47	94	<0.001	<0.001	<0.001
IE_{Ch2}	6	20	2	10	1	20	50	100	<0.001	<0.001	<0.001

Abbreviations: MalExp, Malaria-exposed; Trav, Travelers; Expat, Expatriates; IEs, Infected erythrocytes.

[a] The Fisher's exact test was used.

[b] Determinations were done in children n=28

[c] Determinations were done in expatriates n=5
