# Supplementary material for: Cytokine and Antibody Responses to Plasmodium falciparum in Naïve Individuals during a First Malaria Episode: Effect of Age and Malaria Exposure
Source: PLoS One. 2013 Feb 21;8(2):e55756. doi: 10.1371/journal.pone.0055756 (PMC3578867; doi:10.1371/journal.pone.0055756)
Supplement: Table S2 — Spearman correlations between cytokines/chemokines and parasitemia. (PDF) [file pone.0055756.s002.pdf]

Table S2. Spearman correlations between cytokines/chemokines and parasitemia

	Children n=45		Travelers n=20		Expatriates n=14		Malaria-exposed n=90	
	rho	*p*	rho	*p*	rho	*p*	rho	*p*
IFN-γ	0.0498	0.7484	0.2784	0.2347	0.7982	**0.0006**	-0.1787	0.0920
IL-4	0.0507	0.7407	0.1899	0.4226	0.5842	**0.0283**	-0.0233	0.8272
IL-10	0.2914	**0.0501**	0.6324	**0.0028**	0.3682	0.1951	0.1064	0.3182
IL-8	-0.0316	0.8370	0.5061	**0.0228**	-0.3682	0.9285	-0.0451	0.6731
IL-6	0.1296	0.3961	0.7277	**0.0003**	0.4124	0.1429	0.0740	0.4880
IL-1β	0.0749	0.6246	0.4873	0.0293	0.6449	0.0128	-0.0441	0.6797
